# Argon Cold Atmospheric Pressure Plasma‐Induced Effects on Glucose

**DOI:** 10.1002/fsn3.70565

**Published:** 2025-07-22

**Authors:** Matteo Colombo, Filippo Fossati, Robert Köhler, Martin Bellmann, Lars ten Bosch, Georg Avramidis, Alessia Candeo, Gianluca Valentini, Christoph Gerhard

**Affiliations:** ^1^ Dipartimento di Fisica Politecnico di Milano Milan Italy; ^2^ Faculty of Engineering and Health University of Applied Sciences and Arts Goettingen Germany; ^3^ Department for Knowledge and Technology Transfer University of Applied Sciences and Arts Hildesheim Germany

**Keywords:** argon, ATR‐FTIR, cold atmospheric pressure plasma, food applications, glucose degradation, XPS

## Abstract

The aim of this study was to evaluate the effects of CAP plasma on glucose. Plasma treatments were performed using an argon‐driven atmospheric pressure plasma device whose discharge environment was initially characterized by optical emission spectroscopy. Each sample was subjected to plasma treatments of increasing duration, up to 16 min. X‐ray photoelectron spectroscopy analysis was performed to determine the atomic concentrations of carbon, nitrogen, and oxygen of the outermost surface and to identify the chemical bonding states, revealing significant oxidation of the surface and minor incorporation of nitrogen. Attenuated total reflectance Fourier‐transform infrared spectra of each sample were recorded, from which the intensity evolution of the relevant bands over time was derived. The IR spectra indicate an opening of the glucopyranose ring in glucose, suggesting its enveloping degradation due to plasma treatment at different exposition durations with CAP plasma. In addition, reactive oxygen and nitrogen species in the plasma discharge caused the formation of C=O as part of carboxylic groups and small amounts of carbon–nitrogen moieties or carbon–carbon double bonds in the plasma‐treated samples.

## Graphical abstract

This study describes the effects of cold atmospheric plasma (CAP) on glucose. Samples were treated for up to 16 min using an argon‐driven plasma device, revealing chemical modifications such as oxidation processes and ring openings on glucose. The results show that reactive plasma species led to the formation of carbonyl groups and possibly to carbon‐nitrogen and carbon–carbon double bonds.

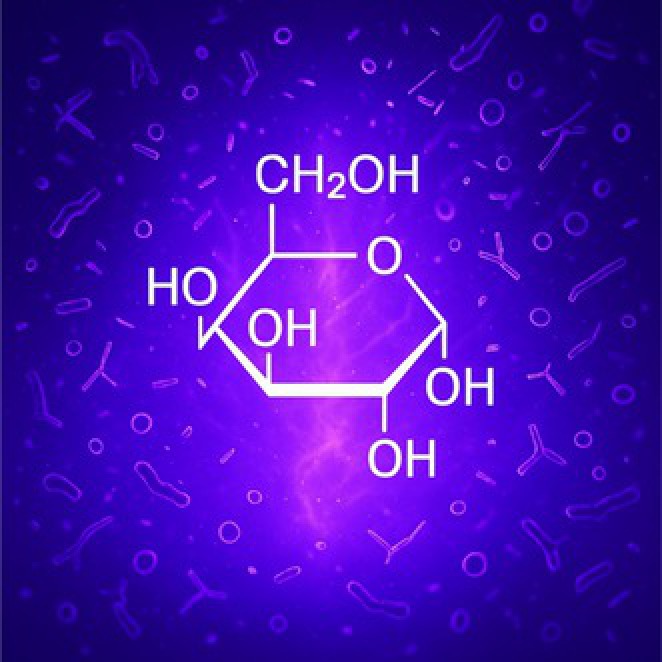

## 1 Introduction

Chemical sterilization and disinfection agents are frequently employed for decontamination purposes in various sectors, including healthcare and the food industry. Despite their ubiquity, there are inherent limitations, including potential health risks, material incompatibilities, and the possibility of contributing to the emergence of antimicrobial resistance. This underscores the necessity for innovative, alternative technologies, such as the utilization of electrical gas discharges for decontamination or disinfection (Das et al. 2024a). Cold atmospheric pressure (CAP) plasma is a partially ionized gas at room temperature and atmospheric pressure, created by applying a strong electric field between two electrodes. This process produces ions, electrons, radical species, and radiation. Among these, radical species (RS) formed during the plasma discharge are highly reactive and are particularly useful for plasma surface treatment, such as polymer activation (e.g., Neto et al. 2020) or pathogen inactivation (e.g., Adesina et al. 2024). CAP plasma operates non‐thermally at atmospheric pressure, making it suitable for integration into production processes in industries such as biomedical, textile, and food processing.

Research on CAP plasma in the food industry has highlighted its potential to be used in the direct decontamination of food packaging, machinery, and food products. Pavlovich et al. (2013) and Miao and Yun (2011) reported reductions of up to 5.5 logCFU/mL in microbial counts on various contaminated surfaces after a few minutes of CAP plasma treatment. Similarly, Gök et al. (2019) showed that CAP plasma effectively inactivated bacteria of Staphylococcus and Listeria strains on dried beef, while Filipic et al. (2020) extended these findings to include viruses. The primary mechanism involves RS and UV light disrupting microbial intracellular components, thereby contributing to food preservation (Majumdar et al. 2018). Das et al. (2024b) characterized the reactive species and inactivation mechanisms of an Ar‐driven CAPJ discharge, demonstrating a reduction of over 6 logs in the number of *Escherichia coli* and *Staphylococcus aureus* within 60 and 120 s of CAPJ exposure, respectively. Further, Dimitrakellis et al. (2021) found that CAP plasma treatments lasting 10–15 min could significantly extend the shelf life of various foods. However, prolonged CAP plasma exposure may degrade compounds in plants that are beneficial to health. Munekata et al. emphasized the need to balance decontamination efficacy with preservation of bioactive compounds and maintenance of nutritional quality by carefully selecting plasma treatment parameters (Munekata et al. 2020). Kim et al. (2024) inoculated fried fish paste with *S. aureus* and *S. typhimurium* and achieved a reduction of up to 1.13 log CFU/g of both bacteria strains using a nitrogen‐driven floating electrode–dielectric barrier discharge (FE‐DBD) within a treatment duration of 60 min. While most literature is primarily focused on the effectiveness of CAP plasma treatment for pathogens decontamination, further investigation into its impact on organic molecules that constitute food, such as proteins, carbohydrates, lipids, and vitamins, is essential. Scheglov et al. (2018) employed XPS and ATR‐FTIR to analyze samples of the amino acids proline and hydroxyproline exposed up to 180 s to a DBD operated with ambient air and observed the attachment of new NO_3_ and NO_2_ groups. In addition, proline was found to be more susceptible to ring opening, fragmentation of the molecule, and the addition of C─OH and C=O groups. Kheto et al. (2023) studied the effect of CAP plasma treatment on guar seed flour (GSF), a protein‐based powder, by Fourier‐transform infrared spectroscopy (FTIR). Low‐intensity treatments (< 10 min) did not affect the nutritional and anti‐nutritional properties of GSF, whereas high‐intensity treatments (10–20 min) did alter the protein structure and oxidation rate of the amino acid chain. Regarding lipids, Pérez‐Andrés et al. (2020) observed a decrease in lipid concentrations after CAP plasma treatment, attributed to the hydrogenation of double bonds in unsaturated fatty acids caused by reactive oxygen species (ROS) in the plasma discharge. If the plasma treatment can be considered beneficial for trans fatty acid diminishment, negative effects arose due to plasma‐induced oxidation of lipids, which affected the acceptability and shelf life of food (Gavahian et al. 2018). Research on vitamins has predominantly focused on ascorbic acid, with the majority of studies reporting no significant reduction in ascorbic acid levels following plasma exposure to kiwifruit and radish sprouts.

(Oh et al. 2017; Ramazzina et al. 2015). The largest recorded reduction of vitamin C was reported by Wang et al. (2012) for cut fruits and vegetables, which amounted to 4%. Sonkar et al. (2023) treated kodo millet starch (KMS) with cold plasma for up to 30 min, finding no discernible changes in the IR spectrum, indicating that its structure remained intact. However, changes in the microstructure of KMS were observed due to fragmentation, agglomeration, and cross‐linking. Additionally, a separate study on bread (Starek‐Wójcicka et al. 2022) revealed that no mesophilic bacteria or fungi were present after ten minutes of CAP plasma exposure, whereas the texture of the bread altered during storage following the treatment, which was attributed to moisture loss during the plasma exposure.

In previous work (Hauswirth et al. 2022), we examined the impact of CAP plasma treatment, generated by a homemade direct dielectric barrier discharge source (introduced in (Brückner et al. 2011)) operated with argon and synthetic air mixture, on glucose and sucrose. The plasma treatments were conducted for up to 12 min, and spectroscopic analysis was performed using both ATR‐FTIR spectroscopy and XPS. The emergence of new bands, which were attributed to double‐bonded carbon or carboxyl groups, was observed. The results indicated that short treatment times caused minimal impact on saccharose and glucose. However, further studies are needed to better understand CAP plasma's effects on food nutrients and assess its safety for food applications. The current study aimed to evaluate the impact of a CAP plasma source (Bellmann et al. 2024) using pure argon as the working gas on the molecular structure and stoichiometry of glucose as a model substance. The goal was to compare these findings with existing literature and expand the available data. XPS was employed to determine the atomic concentrations of carbon (C), oxygen (O) and nitrogen (N) prior to and following plasma exposure, in addition to the chemical bonding states. ATR‐FTIR was utilized to detect the formation of new bonds or the rupture of existing ones, as well as chemical shifts or alterations in the molecular structure.

## 2 Materials and Methods

### 2.1 Sample Preparation

Glucose was purchased by Sigma Aldrich (Sigma‐Aldrich Chemie GmbH, Taufkirchen, Germany) available in a fragmented crystal powder form. The samples were prepared following this procedure: The first step consisted of a wet‐milling of the carbohydrate with a mixer mill (Retsch Mixer Mill MM200). 250 mg of the nutrient were poured into the grinding jar, acrylic grinding balls (0.4 mm glass beads) were added until 2/3 of the jar was filled, and isopropyl alcohol was used to fill the empty gaps in between the grinding balls. The mixer was activated for 10 min at a frequency of 60 Hz. At the end of the procedure, a compact slurry was obtained, which was separated from the glass beads with isopropanol. The solution was collected with a 1000 μL micropipette and stored in a sealed glass container. Subsequently, microscope glass slides (76 × 26 mm^2^) were placed on a pre‐heated hot plate at 50°C. The slurry was pipetted multiple times onto the glass slide with a 10 μL micropipette until a 5 mm diameter circular area with a smooth surface was obtained in order to ensure a uniform plasma treatment.

### 2.2 Plasma Treatment

**FIGURE 1:**
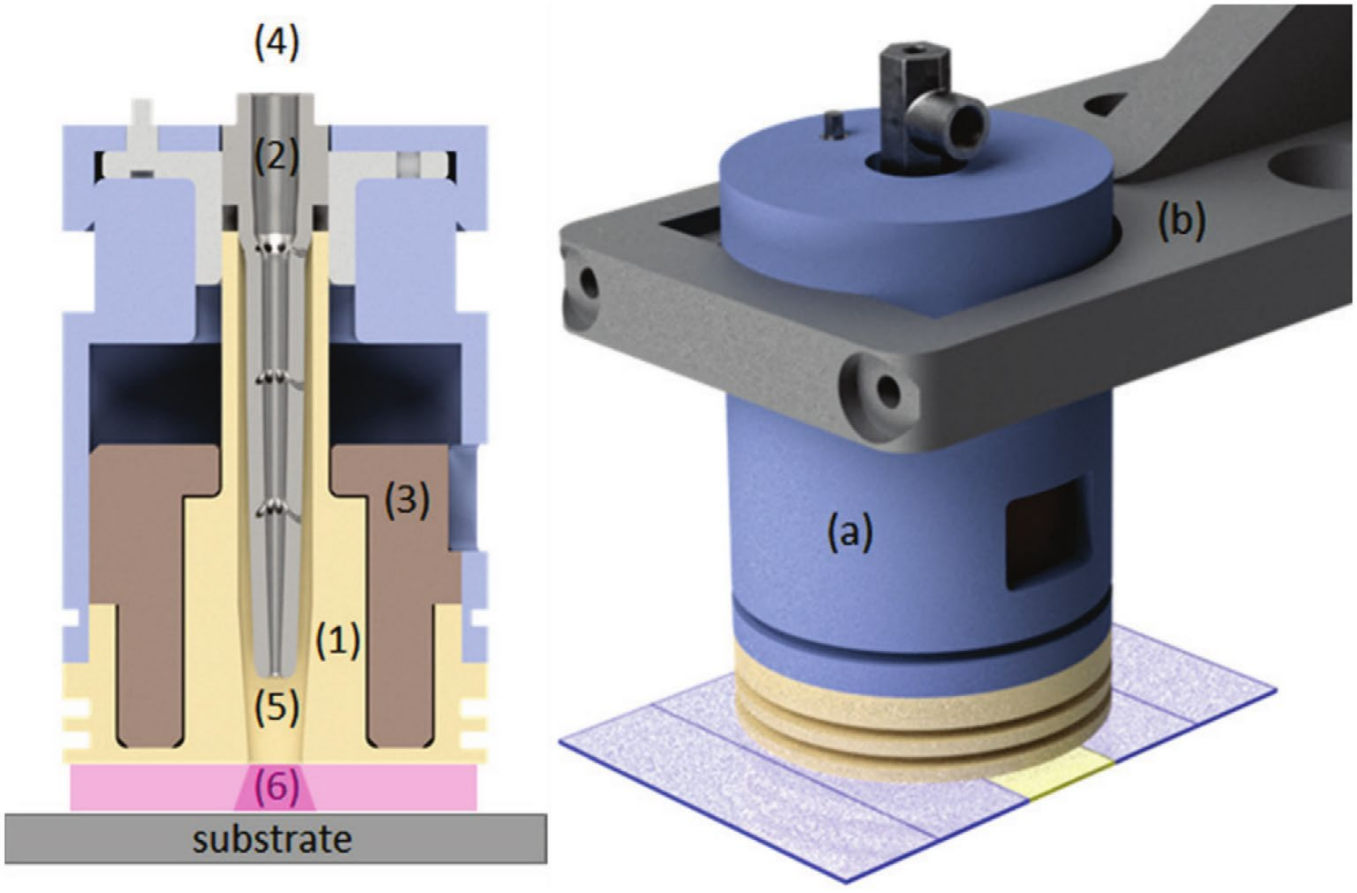
Cross section of the used plasma device (left panel) and experimental setup (right panel), (adapted from (Bellmann et al. 2024)).

A plasma jet‐system introduced in detail in (Bellmann et al. 2024) was used as plasma device in order to treat the samples. The schematic of the plasma source is presented in the left panel of Figure 1. The cylindrical symmetry of the electrodes generates a jet‐induced surface sliding discharge (SSD), which is dielectrically hindered on the lateral surface by an aluminum‐oxide ceramic (1). The jet discharge is generated between the titanium high‐voltage electrode (2) and the dielectrically hindered copper heat sink (3), which acts as ground electrode. Argon (purity ≥ 99.996%, purchased by Linde GmbH) as process gas flows from the upper inlet (4) into the discharge chamber (5) and exits at the lower outlet (6). This results in a cold plasma “gas cushion” between the sample surface and plasma source, with a temperature of about 60°C and a nominal electron energy of approx. 1 eV. However, species of notably higher energy most probably occur in such a plasma. This includes electrons of higher energy due to the plasma‐inherent electron energy distribution (Gudmundsson 2001), metastable argon atom states carrying energy of some tens of eV (Piper 1974; Marchand and Cardinal 1979); and UV photons emitted by the plasma as indicated by the secondary x‐axis in Figure 4.

The experimental setup is depicted in the right panel of Figure 1. The plasma source (a) is positioned on a holding module (b) welded to a translating table (“ISEL Gantry OverHead M20” – Isel Germany AG). Argon as process gas is injected from the inlet and the resulting plasma jet is expelled from the bottom outlet, flowing onto the sample (highlighted in yellow). The sample must be placed in correspondence of the central bottom bore, where the indirect jet discharge occurs. The plasma source was positioned 2 mm above the sample, the argon flow rate was adjusted to 15 sL/min^−1^. The substrate (yellow) was positioned in the middle between four dummy slides (blue) directly under the plasma source. The dummy slides are necessary to provide the discharge with a flat surface in order to prevent potential turbulence caused by substrate edges.

**FIGURE 2:**
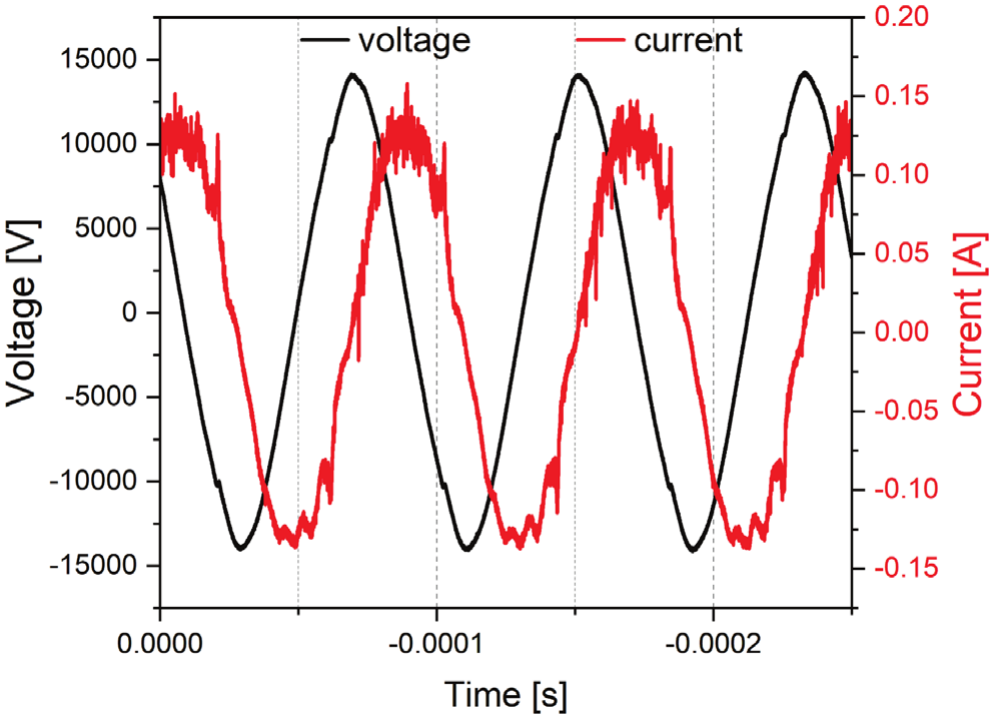
Voltage–current characteristic of the used plasma source.

A high voltage generator (“HV‐X20” – Tantec A/S) and a transformer (“HT‐X1‐34” – Tantec A/S) powered the plasma device by sinusoidal pulses at a frequency of ≈12 kHz and a voltage of approx. 14 kV (peak), with a plasma power density of approx. 3 W/cm^2^ (see Figure 2).

Treatment times were appropriately chosen, so that no visible alteration occurred on the surface for the maximum treatment time (e.g., signs of thermal impact). The treatment durations for glucose were indeed 0, 1, 2, 4, and 16 min. In order to minimize thermal impact for treatment durations > 1 min, after every 60 s of treatment, the source was switched off for 15 s, allowing the sample to cool down, and afterward the treatment was continued.

### 2.3 Optical Emission Spectroscopy

**FIGURE 3:**
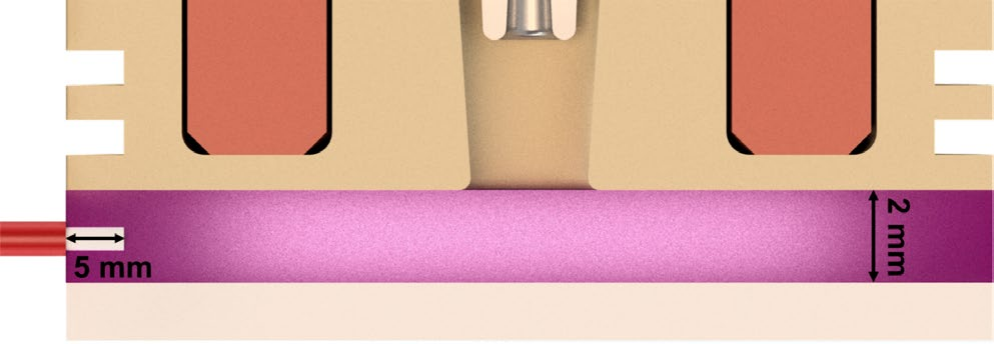
Positioning of the optical fiber during OES measurements (sketch not true to scale).

The actuflasma was thus characterized via optical emission spectroscopy (OES). Measurements were carried out using a fiber‐coupled UV‐VIS‐NIR spectrometer (MUT GmbH, model Tristan light). OES measurements were performed without any sample using the same plasma parameters used for the treatments; the measurement encompassed a spectral range extending from 200 to 1000 nm, and the step size was approximately 0.6 nm. Depending on the wavelength under investigation, the spectrometer's resolution ranges from 0.5 to 2.0 nm. The optical fiber was placed horizontally, as this was the position that provided the best signal‐to‐noise ratio for the ROI of the spectrum. The bare fiber end tip (not including thewithout any protective cladding) protruded vertically, centered approximately 5 mm in the radial direction into the plasma zone. This ensured that, with the exception of a 5 mm wide ring segment in the edge area, the entire radiation emission of the plasma region generated by the source could be detected (see Figure 3).

### 2.4 X‐ray Photoelectron Spectroscopy

X‐ray photoelectron spectroscopy (XPS) measurements were performed on glucose in plasma‐treated and untreated (raw) states. To determine the chemical binding state, XPS measurements were performed on a commercial XPS apparatus (PHI 5000 Versa Probe II from ULVAC‐PHI, Chigasaki, Japan) using monochromatic Al‐K radiation with a photon energy of 1486.6 eV. Detailed spectra of carbon (C1s) and nitrogen (N1s) were collected at a constant electron take‐off angle of 45° over a total scanned area size of 200 × 1400 μm^2^ with a spot size of 100 μm and an X‐ray power of 100 W. The pass energy and step size were set to 46.95 and 0.1 eV, respectively. The spectrometer was calibrated to the reference lines of copper and gold at 932.62 and 83.9 eV, respectively. The minimum detector resolution measured with a pass energy of 46.95 eV at the silver peak Ag (3d5/2) was 0.79 eV. In the case of the survey spectra, the same spot size was used, whereas the pass energy and step size were set to 187.85 and 0.4 eV, respectively. During measurement, the temperature was kept constant at room temperature, and the base pressure was 2 × 10^−6^ Pa. In order to avoid charging effects, the measurements were carried out with the neutralization of sample charging. For each sample, three measurements were performed (*n* = 3). Data processing was performed using MultiPak software (Ulvac‐phi Inc.); a Shirley background spectrum was subtracted prior to peak fitting analysis, separation was performed using Voigt profiles (lower limit at 70% to 100% Gaussian), and the N1s spectra were smoothed using a Savitzky–Golay filter.

### 2.5 Fourier‐Transform Infrared Spectroscopy

Infrared spectra were recorded for each sample prior to and following plasma treatment, spanning the range from 4000 cm^−1^ to 400 cm^−1^. To this purpose, a commercial Fourier‐transform infrared (FTIR) spectrometer in attenuated total reflection (ATR) mode (Perkin‐Elmer Frontier FTIR spectrometer) was employed. The reference spectrum was initially obtained with the ATR module (Golden Gate Diamond ATR with ZnSe lens) in situ. Subsequently, the samples prepared on microscope slides were flipped on the ATR crystal. By applying appropriate contact pressure, it was ensured that the samples were firmly attached to the crystal. Subsequently, the particular IR spectrum was recorded by averaging 64 subsequent spectra. The software automatically applied ATR and baseline corrections. To facilitate comparison of different spectra of the same nutrient, a normalization procedure was performed: each intensity value *I*_*v*_ corresponding to a wavenumber *v* was divided by the Euclidean norm of the whole spectrum:

(1) $$I_{\bar{\nu },\;\text{normalised}}=\frac{I_{\bar{\nu }}}{\sqrt{\sum {I_{\bar{\nu }}}^{2}}}$$

This resulted in a normalized spectrum such that:

(2) $$\sum_{\bar{\nu }}{I_{\bar{\nu },\;\text{normalised}}}^{2}=1.$$

## 3 Results and Discussion

### 3.1 Characterization of Reactive Species in the Plasma Discharge by OES

**FIGURE 4:**
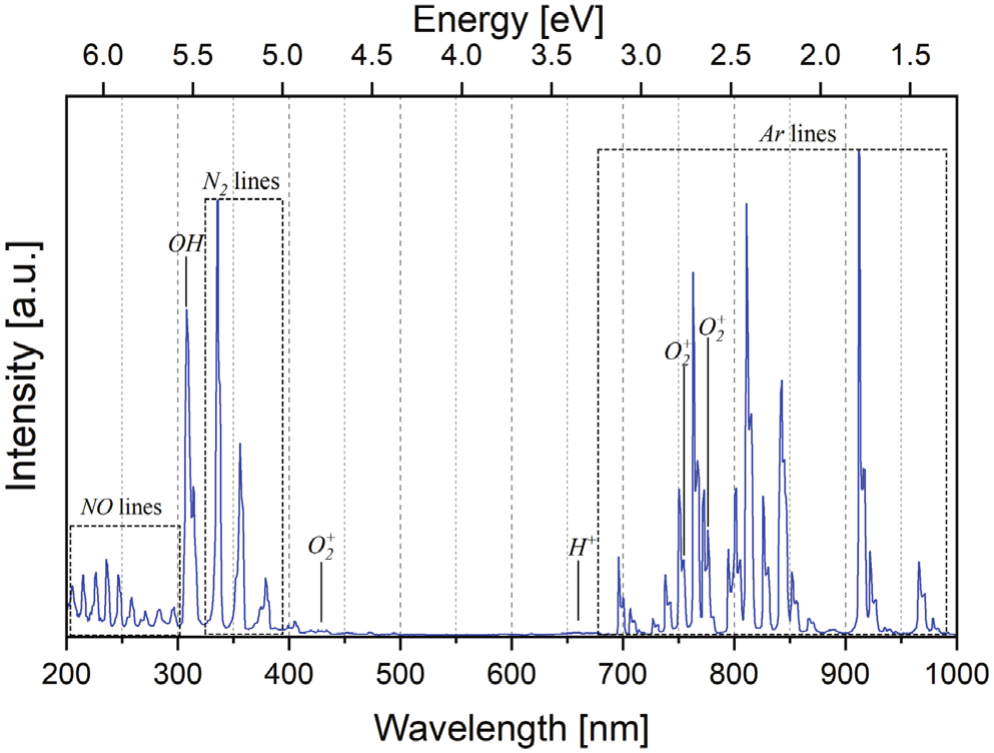
Optical emission spectrum of the used plasma.

A typical OES spectrum of the argon plasma used in this work is presented in Figure 4. Here, characteristic lines of the used process gas, argon, as well as several peaks related to possible transitions between the energy levels of the excited chemical species of the plasma can be identified.

Most of the intense lines observed between 700 and 1000 nm can be attributed to excited argon states (Antipov et al. 2020; Barkhordari et al. 2017; Giannakaris et al. 2023). Moreover, several peaks of the main reactive nitrogen species (RNS) and reactive oxygen species (ROS) are clearly visible. The nitrogen lines between 200 and 300 nm are related to NO radicals (Barkhordari et al. 2017), originating from radiative transitions (A‐X transitions) between NO states excited by impact with N_2_ molecules in metastable states. The lines between 350 and 450 nm are related to N_2_ and N_2_^+^ C‐B transitions, whereas the intense line at 307.8 nm is related to ·OH radicals (Antipov et al. 2020). The presence of this highly reactive species can be explained by the humidity of air, which is also confirmed by the weak H^+^ line at 656.3 nm, aka the Balmer H‐α line. Possible oxygen lines can be found at 774.4, 752.9, 470.5, 434.2, 353.3, and 343.2 nm (Paatre Shashikanthalu et al. 2020). These—partially weak—peaks can be related to ROS species such as O_2_^+^ and O_3_. Hence, several RNS and ROS species can be found in the used plasma, and plasma‐induced reactions in treated starch samples may be caused by ·OH, N_2_, NO radicals, and N_2_^+^ ions. It should finally be taken into consideration that typically ROS species feature higher reactivity in comparison to RNS.

### 3.2 XPS Analysis of Untreated and Plasma Exposed Glucose

A comparative analysis of the XPS spectra of raw glucose and plasma‐treated glucose reveals only marginal alterations in the atomic concentration of carbon and oxygen. However, a notable presence/increase in nitrogen can be discerned, although the absolute quantities of nitrogen remain low (see Table 1).

**TABLE 1:** Atomic concentration of C, O, N before and after plasma exposition at different time scales (*n* = 3).

t_P_ (min)	0	1	2	4	16
at% C	58.2 ± 1.8	58.9± 0.2	61.2 ± 0.4	60.9 ± 0.3	60.2 ± 0.4
at% O	40.9 ± 2.1	40.3± 0.3	37.7 ± 0.3	38.0 ± 0.5	39.1 ± 0.4
at% N	0.2 ± 0.2	0.8 ± 0.2	1.1 ± 0.1	1.2 ± 0.2	0.7 ± 0.2

**FIGURE 5:**
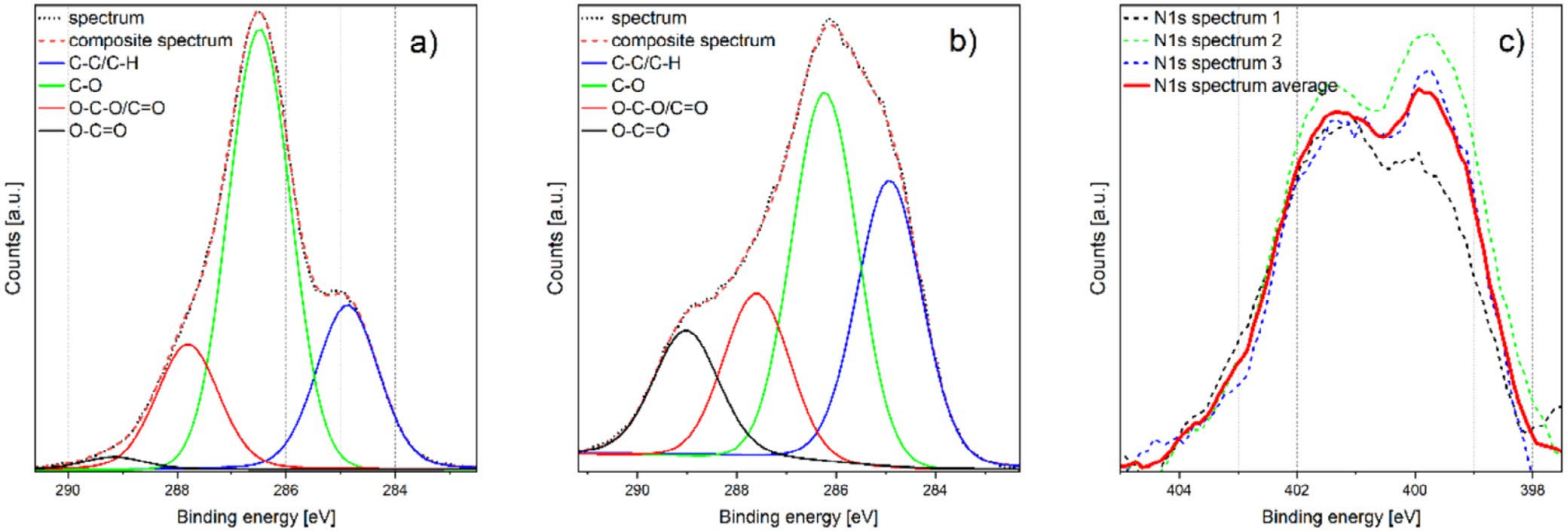
Deconvoluted C1s detail spectra for untreated (a), 16 min plasma‐exposed specimen (b) and N1s spectra (smoothed) of 16 min plasma‐exposed specimen (c).

Figure 5 illustrates the deconvoluted C1s spectra of unmodified glucose (Figure 5a) and a sample exposed to plasma for 16 min (Figure 5b). It is evident that there has been a reduction in the intensity of the C‐O signal (286.5 eV), whereas for the plasma‐exposed samples, a new peak is observed at ≈289 eV, which is assigned to carboxylic moieties (O‐C=O). Given the low concentrations detected (see Table 1), the analysis did not include carbon‐nitrogen bonds (≈286 eV for C=N, ≈287 eV for C‐N (D'Anna et al. 1999)) in the deconvolution of the C1s spectra, as they are almost entirely obscured by superposition with dominant C‐C‐ or C‐O‐moieties in the recorded spectra. However, the position of the center of the nitrogen peak at the N1s spectra for the plasma‐treated samples (at ≈400 eV) allows the presence of nitro and nitroxy groups (405.7 and 408.3 eV) as well as nitrite and nitrate components (403.9 and 407.2 eV) in significant moieties to be excluded on the basis of the observed data (Figure 5c). Conversely, the presence of functional groups such as (among others) amines, amides, or cyanates (Mohtasebi et al. 2016; Tardio et al. 2019) within this spectral range cannot be discounted.

In order to obtain an overview of the distribution of carbon bonds with increasing exposure time, C1s detail spectra were generated for samples exposed to the plasma for 1, 2, 4, and 16 min, as well as for untreated samples. The spectra were then deconvoluted according to C‐C and carbon–oxygen bonds. The distribution of carbon bonds (C‐C/C‐H, C‐O, O‐C‐O/C=O, O‐C=O) over the treatment time is presented in Table 2. It can be observed that the proportion of O‐C‐O/C=O bonds remains largely unaltered, whereas the proportions of C‐C/C‐H bonds, C‐O, and carboxy groups undergo a notable change.

**TABLE 2:** Atomic percent distribution (at%) of carbon bonds with enveloping exposition duration by deconvolution of C1s spectra (*n* = 3, whereas *n* = 2 for 0 min).

t_P_ (min)	0	1	2	4	16
at% C‐C/C‐H	24.4 ± 1.4	29.2 ± 0.6	36.3 ± 1.6	36.9 ± 2.1	34.1 ± 2.8
at% C‐O	57.8 ± 0.8	45.6 ± 0.5	38.3 ± 0.6	36.5 ± 1.4	36.7 ± 1.7
at% O‐C‐O/C=O	15.8 ± 0.7	16.8 ± 0.1	15.9 ± 0.5	15.5 ± 0.8	16.3 ± 1.0
at% O‐C=O	2.1 ± 0.1	8.4 ± 0.1	9.5 ± 0.6	11.1 ± 0.2	12.9 ± 0.5

**FIGURE 6:**
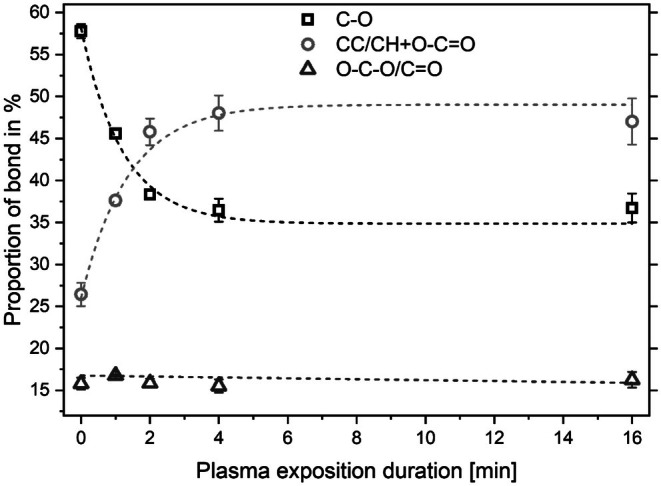
Evolution of carbon–oxygen moieties with elapsed plasma exposition duration.

Figure 6 illustrates the time‐dependent evolution of the deconvoluted carbon bonds of the C1s signal. A reduction in the proportion of simple oxygen–carbon bonds is observed, accompanied by a simultaneous and quantitatively equivalent increase in the summed proportion of carboxyl groups and C‐C/C‐H bonds. The O/C ratio remains approximately constant. This observation can be explained by a partial reduction of C‐O bonds with the simultaneous oxidation of some C‐O moieties to O‐C=O bonds.

### 3.3 ATR‐FTIR Analysis

**FIGURE 7:**
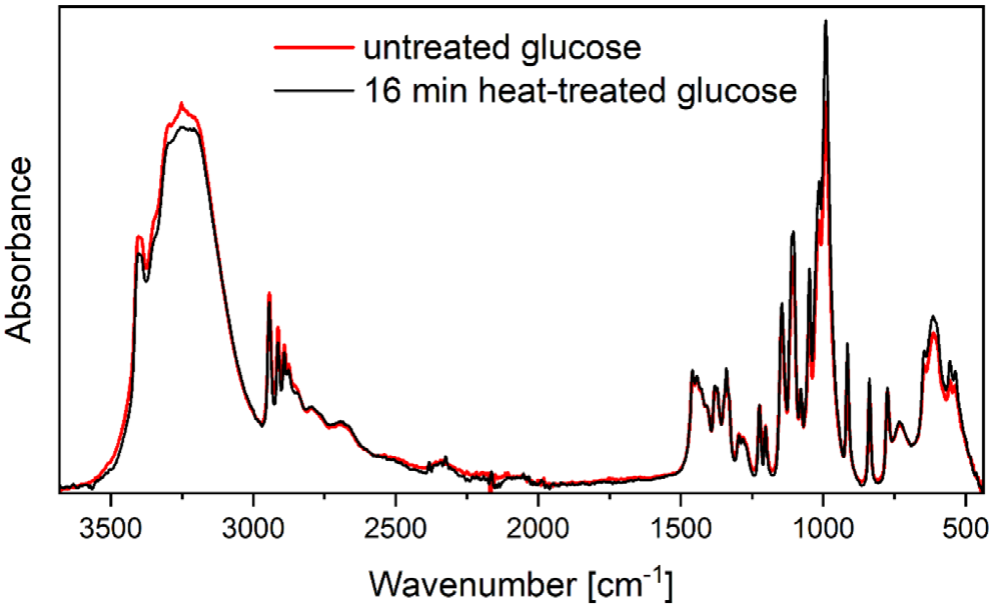
Comparison between the ATR‐FTIR spectra of 16 min heat‐treated glucose at 60°C (black line) and the reference (red line).

In order to demonstrate that any differences observed in the FTIR spectra of a plasma‐treated samples in comparison to the untreated one are attributable to the reactive species present in the plasma, it was first necessary to investigate the effect of temperature. To this end, a specimen was positioned on a heat plate at 60°C for 16 min, which represents the longest plasma exposure duration and the highest temperature recorded during plasma exposure, respectively. The surface temperature of the samples was measured to confirm that the target temperature of 60°C was reached. An ATR‐FTIR spectrum was obtained following a 2 min cooling time at ambient temperature, which was the average time elapsed between the conclusion of a plasma treatment and the corresponding ATR‐FTIR measurement. The comparison of ATR‐FTIR spectra of the reference and 16 min heat‐treated glucose (Figure 7) indicates minimal spectral differences. These minor deviations are considered to represent random errors during the measurement procedure, variations in surface temperature, or sample handling.

**FIGURE 8:**
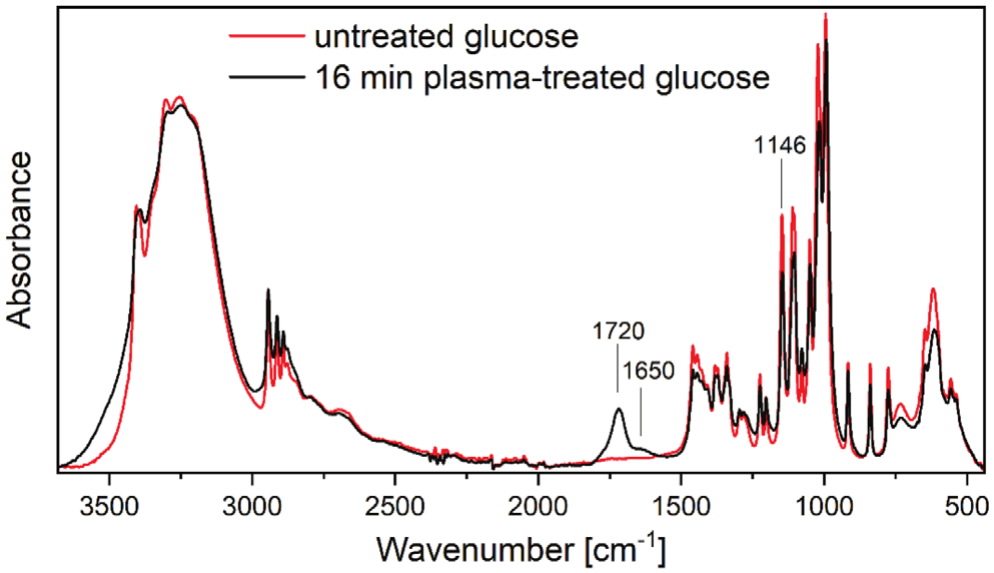
ATR‐FTIR spectra of glucose in untreated state (red line) and after plasma exposition for 16 min (black line).

The absorption bands of glucose were assigned by comparing their spectrum with the existing literature on vibrational spectra in carbohydrates by Wiercigroch et al. (2017). The specific chemical bond associated with each band was determined by analyzing the three‐dimensional projections of glucose (Brizuela et al. 2012). A comparison of the IR spectra of plasma‐treated samples (16 min) with those of the untreated samples is presented in Figure 8. The broad band associated with hydrogen bonds, spanning the range of 3700–2700 cm^−1^, displays a widening, indicative of a potential reduction in crystallinity or an increase in hydroxyl groups. This is corroborated by the observed increase in the height of the 43O‐45H peak, situated at approximately 1200 cm^−1^. With regard to the CH_2_‐related bands in the range 1500–1300 cm^−1^, a reduction in peak height was observed. The bands between 1000 and 1160 cm^−1^ can be associated with CO stretching vibrations, which exhibit a notable decrease in the plasma‐exposed samples. The bands below 750 cm^−1^ can be assigned to ring vibrations, which also demonstrate a decrease, suggesting the cleavage of the ring structure in the plasma‐exposed samples. Breaking points of the closed‐ring structure, leading to the formation of a linear molecule, may also be a possible binding site for OH groups, which contribute to the observed wider hydrogen‐bonding band above 3000 cm^−1^ following plasma treatment. Possible degradation products include α‐D‐glucuronic acid, β‐D‐glucuronic acid, D‐gluconic acid, and D‐glucono‐1,5‐lactone. The breaking of CH and CO bonds supports the proposed oxidation pathways (Armstrong et al. 2019). The CH and CH_2_ peaks between 3000 cm^−1^ and 2800 cm^−1^ were not analyzed because their signal is superimposed by the broad OH band between 3500 cm^−1^ and 3000 cm^−1^.

**FIGURE 9:**
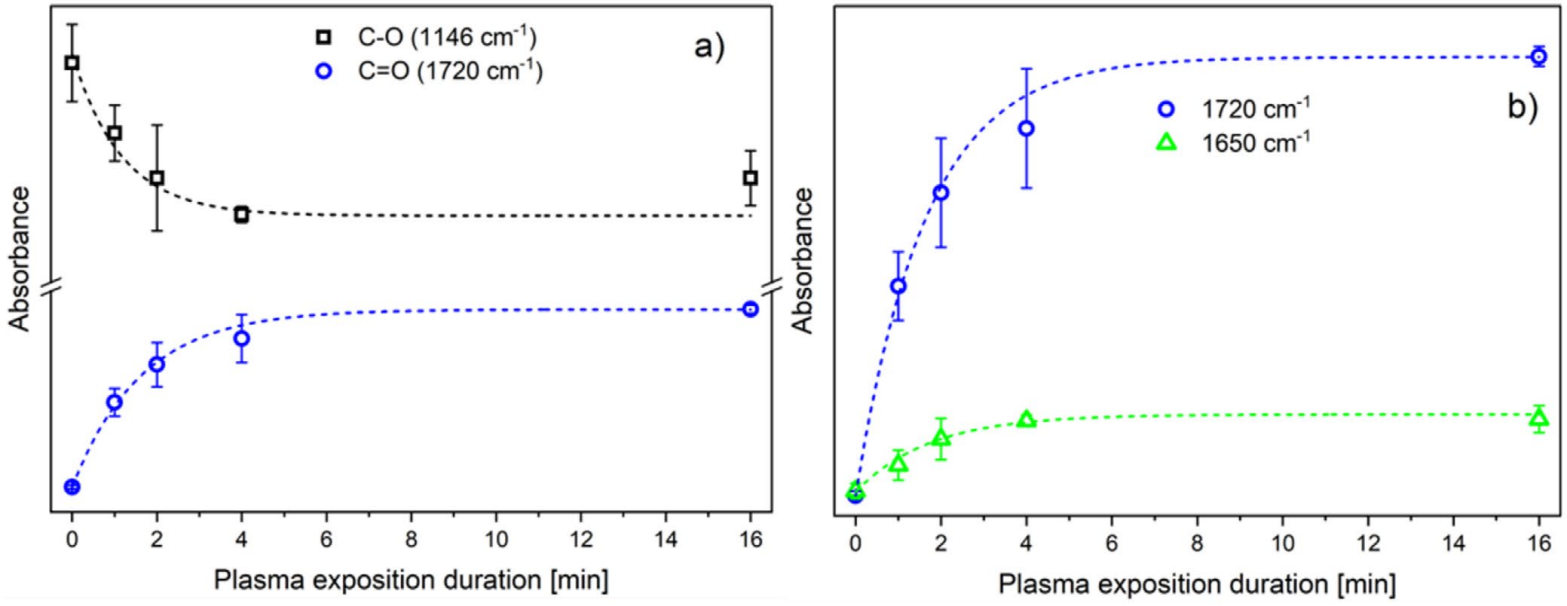
Height evolution of peaks ascribed to C–O (≈1150 cm^−1^), C=O (≈1720 cm^−1^), and at 1650 cm^−1^ with increasing plasma exposition duration.

As illustrated in Figure 9a, the emergence of a new band at approximately 1720 cm^−1^ is accompanied by a temporal increase in peak height and a simultaneous decrease in the band at approximately 1150 cm^−1^, with increasing plasma exposure time. This reflects the general trend of these bands. The band at 1720 cm^−1^ can be assigned to C=O, while the band at 1150 cm^−1^ corresponds to C‐O components. The results of the XPS analysis indicate that the formed C=O bonds are part of the newly formed carboxyl groups (see Figure 6). This conclusion is also corroborated by the results of previous studies conducted with diverse plasma sources and process gases (e.g., (Hauswirth et al. 2022)).

Figure 9b depicts the emergence of a supplementary new band at approximately 1650 cm^−1^ (together with the 1720 cm^−1^ band at reduced y‐scaling) which can also be attributed to carbonyl (C=O) stretching. Nevertheless, C=C stretching vibrations may occur within the range of 1620 to 1680 cm^−1^ (Reis et al. 2006). Although the FTIR band at 1650 cm^−1^ is typically not associated with carbon–carbon double bonds (C=C), C=C bonds have been observed to form following the exposure of glucose and cellobiose to argon plasma (Klarhofer et al. 2010). Moreover, the 1650 cm^−1^ band can occur in specific contexts with the amide I band and other amide‐containing compounds (Ozaki et al. 1997), which correlates with the appearance of nitrogen in the XPS analysis (see Table 1 and Figure 5). It can therefore be surmised that C=C or C=N bonds may contribute to bands in this region, although it is more probable that the predominant contribution is due to C=O groups.

The potential occurrence of C=N or C=C compounds following plasma exposure to glucose‐containing foods (or carbohydrates in general) could be associated with adverse health effects. It is important to note, however, that the proportions of these compounds will only occur to a small extent, given that the degradation of toxic substances in the context of plasma exposure occurs at short exposition times. Considering the degradation kinetics of mycotoxins reported by ten Bosch et al. (2017) reveals that the majority of mycotoxins were significantly degraded after approximately 30 s of plasma exposition, even when embedded in a rice extract, although the degradation rate was then less pronounced. Park et al. found similar degradation kinetics of mycotoxins when using a plasma jet system also operated with argon (Park et al. 2007). Taking into account the degradation rates of mycotoxins reported in ten Bosch et al. and Park et al. and the increase in the 1650 cm^−1^ band which may represent C=N or C=C compounds in this study (Figure 9), exposure times of less than 1 min are recommended. This enables a significant proportion of the contaminants to be degraded without causing a notable increase in the 1650 cm^−1^ band (or the amount of possible C=N or C=C). Additionally, only minimal penetration depths were observed during plasma treatment of biological substrates. As reported by Král et al. (2015), the effective penetration depth of plasma in wood is only a few hundred nanometers, as determined by depth‐resolved XPS analysis data. Consequently, it can be postulated that the absolute proportions of the aforementioned (possibly hazardous) compounds in a brief plasma treatment of carbohydrate‐containing foods will be insignificant compared to reported harmful concentrations (Govindaraju et al. 2024).

## 4 Conclusions

This study presents an analysis of the oxidative effects on glucose induced by an argon‐driven cold atmospheric pressure plasma device. The analysis encompasses both structural and compositional changes. Optical emission spectroscopy was employed to identify a spectrum of reactive nitrogen species (RNS) and reactive oxygen species (ROS), including NO, N_2_^+^, and OH, which drive the oxidative processes. The findings are corroborated by shifts in the XPS spectra and the emergence of new bands in the FTIR spectra. Notably, a discernible decline in the intensity of the C‐O bond was observed alongside the emergence of peaks indicative of carboxyl groups (O‐C=O). The plasma exposure results in the oxidation of C‐O bonds, with the presence of potentially harmful bonds such as C=N or C=C increasing only slightly. The presented process enables the safe modification of food surfaces, which can counteract microbial growth or degrade contaminants such as mycotoxins without introducing harmful residues. The minimal penetration depth of the plasma and the relatively short exposure times for decontamination purposes also suggest that the process can be used on an industrial scale. Future research should focus on optimizing the plasma parameters to ensure consistency and scalability, and to evaluate the long‐term stability and nutritional quality of plasma‐treated foods.

## Author Contributions

**Matteo Colombo:** data curation (equal), formal analysis (equal), investigation (lead), validation (equal), visualization (supporting), writing – original draft (equal), writing – review and editing (supporting). **Filippo Fossati:** formal analysis (equal), investigation (equal), validation (equal), visualization (supporting), writing – review and editing (supporting). **Robert Köhler:** data curation (equal), formal analysis (equal), investigation (supporting), methodology (equal), supervision (supporting), validation (equal), writing – original draft (supporting), writing – review and editing (supporting). **Martin Bellmann:** investigation (supporting), methodology (equal), supervision (supporting), validation (supporting), visualization (supporting), writing – original draft (supporting), writing – review and editing (supporting). **Georg Avramidis:** conceptualization (equal), data curation (equal), formal analysis (equal), methodology (equal), supervision (supporting), validation (supporting), visualization (lead), writing – original draft (equal), writing – review and editing (lead). **Alessia Candeo:** formal analysis (equal), methodology (supporting), supervision (equal), validation (equal), writing – original draft (equal), writing – review and editing (supporting). **Lars ten Bosch:** conceptualization (equal), formal analysis (supporting), methodology (equal), validation (supporting), writing – review and editing (supporting). **Gianluca Valentini:** formal analysis (equal), methodology (supporting), resources (equal), supervision (equal), validation (equal), writing – original draft (supporting), writing – review and editing (supporting). **Christoph Gerhard:** conceptualization (equal), formal analysis (equal), methodology (lead), project administration (lead), resources (lead), supervision (lead), validation (equal), visualization (supporting), writing – original draft (equal), writing – review and editing (lead).

## Conflicts of Interest

The authors declare no conflicts of interest.

## Data Availability

The data that support the findings of this study are available from the corresponding author upon reasonable request.
